# Primary *Klebsiella pneumoniae* Osteomyelitis with Bacteremia and Sepsis in a Patient with Cirrhosis

**DOI:** 10.1155/2018/3183805

**Published:** 2018-10-23

**Authors:** Akshay Khatri, Naga Sasidhar Kanaparthy, Bright Jebaraj Selvaraj, Eunna Cho, Marc Y. El Khoury

**Affiliations:** ^1^Department of Medicine, New York Medical College at Westchester Medical Center, Valhalla, NY 10595, USA; ^2^Division of Infectious Diseases, New York Medical College at Westchester Medical Center, Valhalla, NY 10595, USA

## Abstract

Osteomyelitis is commonly caused by *Staphylococci*, *Streptococci*, *Escherichia coli*, and anaerobes. There have been cases of rare organisms like *Klebsiella pneumoniae* (*Kp*) being initially overlooked as causes of osteomyelitis. We report a case of an elderly cirrhotic adult male transferred for further management of liver failure, who was subsequently diagnosed with *Kp* osteomyelitis and sepsis. He had a history of blunt leg trauma, and MRI of the leg revealed osteomyelitis, with a negative workup for other sources of infection. *Kp* osteomyelitis is reported in less than 100 cases, mainly in pediatric and sickle-cell patients. There are no pathognomonic imaging findings. Lesions may be metastatic, with rapid widespread destruction and exuberant periosteal reaction. *Kp* is a rare, under recognized cause of osteomyelitis in immune-suppressed adults. Given its pathogenicity, early identification is critical.

## 1. Introduction

Osteomyelitis is a progressive infectious process involving various bone components— the periosteum, cortical bone, and medullary cavity. It is characterized by progressive inflammatory destruction of bone, necrosis, and new bone apposition [1].

There are three main types of osteomyelitis, that is, due to local spread from a contiguous source of infection; secondary to vascular insufficiency; and hematogenous osteomyelitis, with seeding of injured bone from bacteria present in the blood [2]. The most common microorganism is *Staphylococcus aureus*. Other microrgansims include coagulase-negative staphylococci, beta-hemolytic streptococci, *Streptococcus viridans*, enterococci, aerobic Gram-negative bacilli (*Pseudomonas* and *Enterobacter* sp. and *Escherichia coli*), anaerobes, and *Mycobacterium tuberculosis* [2, 3]. Hematogenous osteomyelitis is usually monomicrobial, whereas osteomyelitis related to contiguous spread or inoculation may be monomicrobial or polymicrobial [4].

*Klebsiella pneumoniae* (*Kp*) is a rare cause of osteomyelitis, especially in adults. We report a case of *Kp* osteomyelitis and sepsis in a patient with cirrhosis.

## 2. Case Presentation

A 64-year-old African American man, resident of a correctional facility, was transferred from an outside hospital for further management of liver failure. He was initially admitted at an outside hospital when routine blood tests showed leukocyte count 24,000/*µ*L, platelet count 123,000/*µ*L, serum sodium level 127 meq/L, aspartate aminotransferase (AST) 169 U/L, alanine aminotransferase (ALT) 116 U/L, alkaline phosphatase (ALP) 230 U/L, total bilirubin 17.7 mg/dL, and direct bilirubin 13 mg/dL. His past medical and surgical history was significant for decompensated alcohol-induced cirrhosis with untreated chronic hepatitis C; recurrent ascites; hypertension; schizophrenia; cholecystectomy; and appendectomy. He is a current smoker (40 pack-year) but quit drinking alcohol and using intravenous drugs 6 years ago. The patient complained of mild diffuse abdominal pain with several episodes of watery, nonbloody, nonfoul-smelling diarrhea. He denied fevers, chills, vomiting, melena, or hematochezia. He also reported an unintentional weight loss of 18 pounds over the last 6 months. The rest of the review of the system was negative.

On examination, he was alert and oriented, with temperature 37.3 degrees Celsius, pulse 113/min, blood pressure 103/71 mmHg, respiratory rate 18/min, and saturation 96% on room air. He had icteric sclera. Heart and lung exam was normal. Abdomen was mildly distended, nontender, and tympanic with no shifting dullness. Extremities were warm, with left foot and leg-pitting edema and severe tenderness without erythema. On direct questioning, the patient recalled hurting his left leg 8 days prior to admission while removing his boots, followed 3 days later by swelling, pain in the left foot and ankle that progressed to the leg. No skin lesions or wounds were noted.

His admission labs were significant for a leukocyte count of 14,700/*µ*L, hemoglobin 12.1 g/dL, platelet count 137,000/*µ*L, sedimentation rate 20 mm/hr, C-reactive protein 16mg/dL (normal range 0–0.5 mg/dl), AST 161 U/L, ALT 99 U/L, ALP 177 U/L, total bilirubin 15 mg/dL, direct bilirubin 10.7 mg/dL, albumin 1.5 g/dL, and INR 1.54. HIV antibodies were negative. Hepatitis C virus (HCV) RNA was 184,000 copies/mL.

Six hours after admission, the patient developed worsening tachycardia (125/min) with hypotension. He was given 2 liters of intravenous normal saline boluses and started on vancomycin and cefepime empirically. Blood cultures grew a mucoid strain of *K. pneumoniae*, in both aerobic and anaerobic cultures within 8 hours of collection, resistant only to ampicillin. Vancomycin was discontinued, and further serotype testing was not performed at that time.

**Figure 1:**
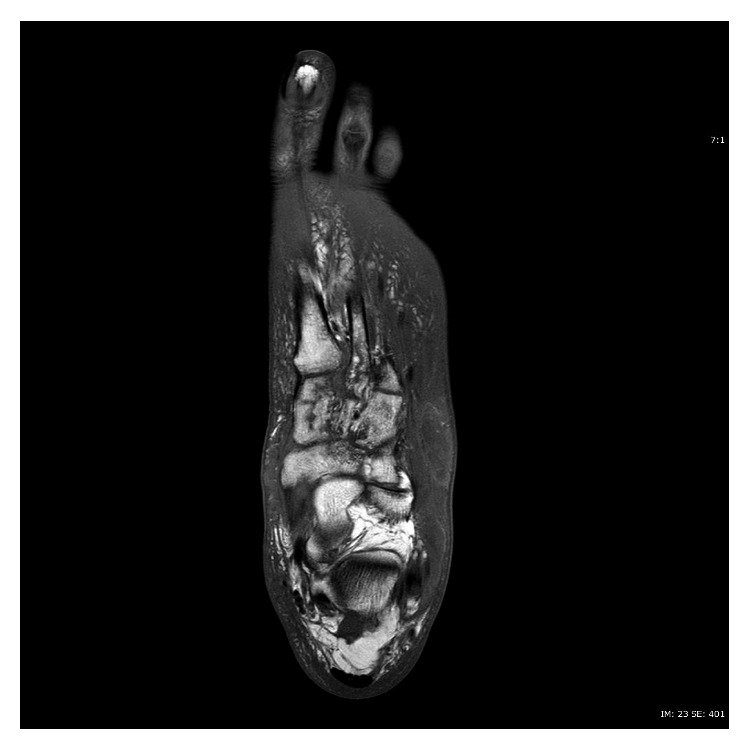
T1-weighted axial image of MRI of left foot without contrast, showing decreased signals (hypointensities) of cuneiforms and metatarsals, reflecting medullary edema and osteomyelitis.

**Figure 2:**
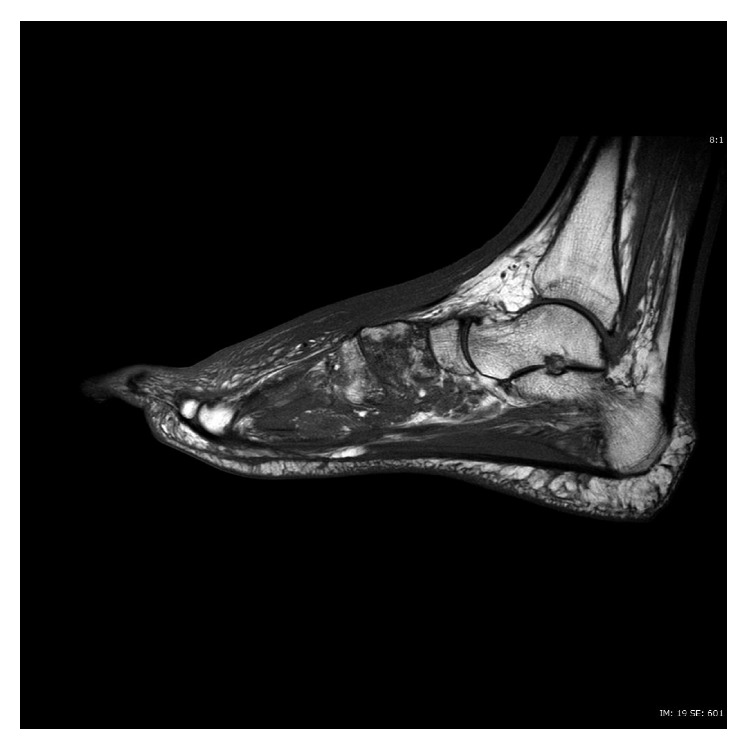
T1-weighted sagittal image of MRI of left foot without contrast, showing medullary edema and osteomyelitis of cuneiforms and metatarsals.

**Figure 3:**
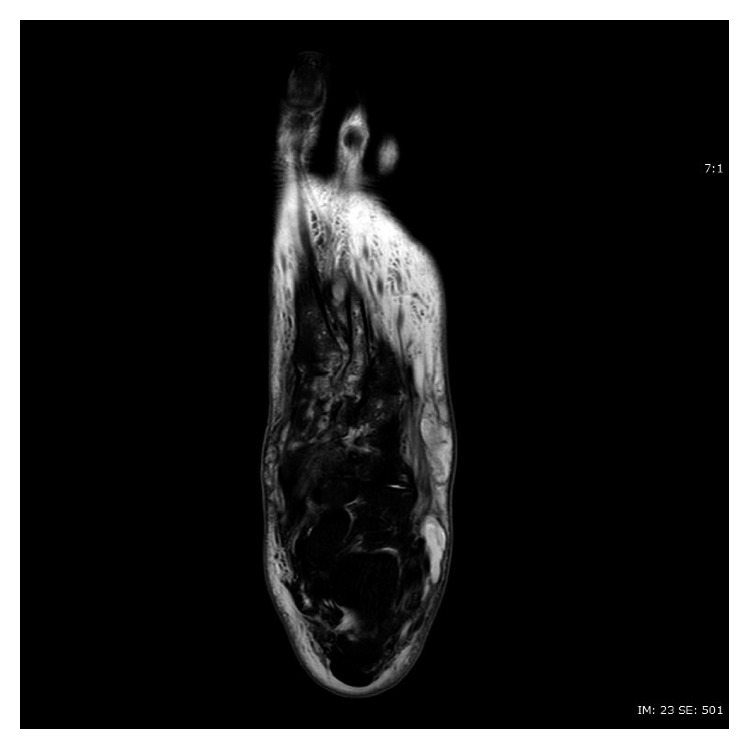
T2-weighted axial image of MRI of left foot without contrast, showing increased signals in soft tissues (compatible with cellulitis), as well as deep-seated multilocular collection at Lisfranc's joints and second metatarsophalangeal joints.

**Figure 4:**
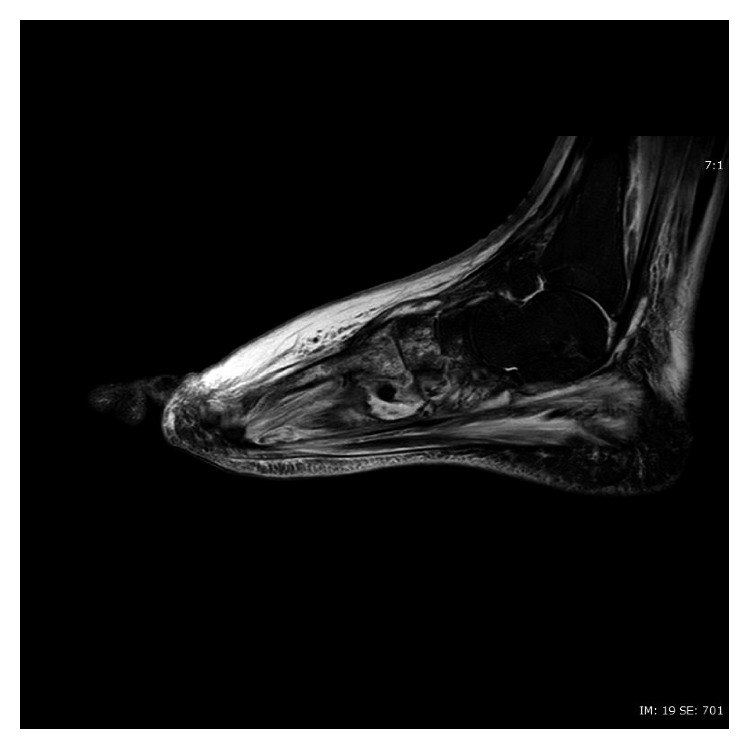
T2-weighted sagittal image of MRI of left foot without contrast, showing cellulitis of soft tissues, as well as deep-seated multilocular collection at Lisfranc's joints and second metatarsophalangeal joints.

A diagnostic paracentesis revealed ascites leukocyte count 358/mL with 66% neutrophils, and peritoneal fluid cultures were negative. A transthoracic echocardiogram was normal. A CT scan of the chest was done showing mild lower lung atelectasis. A triple-phase CT scan of the liver showed an enlarged left hepatic lobe with a shrunken right lobe with cirrhotic morphology, a small right hepatic lobe cyst, and moderate ascites. Magnetic resonance imaging of the left foot and leg without contrast revealed intraosseous abscess in the second metatarsal and marrow edema within the cuneiforms and second through fourth metatarsal bases suggestive of osteomyelitis (Figures 1–3). There was extensive edema within the subcutaneous soft tissues of the leg and dorsum of the foot, with infiltration of subcutaneous fat compatible with cellulitis (Figures 3 and 4).

The patient's hospital course was complicated by encephalopathy requiring transient intubation for airway protection and acute kidney injury secondary to sepsis and hepatorenal syndrome requiring hemodialysis. The patient sepsis and bacteremia resolved; his mental status improved but remained in renal failure. He was discharged to a correctional facility to complete 8 weeks of intravenous ceftazidime.

## 3. Discussion

*Klebsiella pneumoniae* (*Kp*)— also known as Friedlander's bacillus—was first identified in 1882 as an etiological agent in lobar pneumonia [5]. It is one of the few Gram-negative bacilli that can cause primary lobar pneumonia leading in many instances to cavitation [6]. It is the second most common cause of Gram-negative bacteremia [7]. *Kp* is classified into approximately 80 serotypes based on its capsular lipopolysaccharide [8], which is considered a critical virulence factor [9]. *Kp* primarily colonizes the gastrointestinal tract and sometimes the oropharynx [10]. Its presence on the skin is usually considered a transient phenomenon [11] and is sometimes overlooked as a cause of cellulitis or osteomyelitis [12].

Predisposing factors leading to *Kp*-related community-acquired infections include diabetes mellitus, alcoholism, malignancy, or hepatobiliary disease [8, 13]. Diabetes mellitus is the most common predisposing factor as hyperglycemia facilitates the growth of the capsular polysaccharide and promotes resistance to phagocytosis [13].

Nosocomially acquired *Kp* infections are more common in patients with malignancies or following urinary catheterization, chemotherapy, mechanical ventilation, central venous catheter insertion and recent surgery. Antimicrobial resistance is more common, with higher expected morbidity and mortality [13].

In patients with cirrhosis, *Kp* infections are commonly related to spontaneous bacterial peritonitis, urinary tract infections, and community-acquired pneumonia [14]. The incidence of *Kp* infections in cirrhotics is uncommon and variable—one study reported it in 1.9% of patients [15], while another reported it as 8.51% of all organisms isolated [16].

Cellulitis has been diagnosed in 10.5–12.5% of patients with cirrhosis [17, 18]. The exact prevalence of *Kp* osteomyelitis in adults is not known, with axial skeleton involvement (mandible, vertebrae, spine, and pelvic bones) being most commonly reported [19, 20]. *Kp* osteomyelitis was first reported in 1938 in which seven patients with osteomyelitis of the femur, tibia, humerus, or mastoid were described. Five of the seven had a preceding or simultaneous infection with *Kp* at another site [21]. In our case, the patient had no apparent remote source of infection to account for hematogenous seeding; thus, we believe that infection started locally following minor trauma to the skin.

Unlike in adults, *Kp* osteomyelitis and septic arthritis have commonly been reported in children, infants, and neonates [22]. In both adults and children, some cases had a clear source of primary infection [23, 24]; others had no apparent risk factors except minor trauma which was often missed or under reported [25, 26].

*Kp* osteomyelitis is characterized by a tendency to rapid evolution, being multifocal with long bone involvement, widespread destruction, and exuberant periosteal reaction [27]. In addition, in the setting of emerging nosocomially acquired multidrug-resistant *Kp* infection, a microbiologic diagnosis becomes of upmost importance with either bone aspiration or biopsy [6] when blood cultures are negative and when no apparent source is identified [12].

In recent years, new hypervirulent *Kp* strains (in particular, *Kp* serotypes K1 and K2) have been associated with life-threatening infections (bacteremia, liver abscess, and meningitis) with distant metastasis in younger immune-competent hosts [12, 28, 29]. The increased virulence is related to the presence of the capsular (K) antigen, producing a hyperviscous capsule leading to the growth of mucoid colonies on culture plates that resists phagocytosis [12, 30].

Our patient had an infection with a mucoid *Kp* strain sensitive to all antibiotics except ampicillin suggesting a hypervirulent strain. The *Kp* capsular serotype remains unknown as such testing is not routinely performed at our hospital, and it was not requested since the patient had a favorable clinical response to early antimicrobial therapy.

## 4. Conclusion

*Kp* is a rare cause of osteomyelitis in adults, affecting mainly those with underlying immunosuppression. Given its pathogenic nature, early identification and therapy remains a key step in management.
